# Sol–gel based synthesis and biological properties of zinc integrated nano bioglass ceramics for bone tissue regeneration

**DOI:** 10.1007/s10856-020-06478-3

**Published:** 2021-01-20

**Authors:** Pragyan Paramita, Murugesan Ramachandran, Srinivasan Narashiman, Selvamurugan Nagarajan, Dinesh Kumar Sukumar, Tze-Wen Chung, Moorthi Ambigapathi

**Affiliations:** 1 Faculty of Allied Health Sciences, Chettinad Hospital and Research Institute, Chettinad Academy of Research and Education, Kelambakkam, Tamil Nadu 603103 India; 2 Department of Biotechnology, School of Bioengineering, SRM University, Kattankulathur, Tamil Nadu 603203 India; 3 Department of Biomedical Science, Peptide Biochemistry, Chosun University, Gwangju, 61452 Republic of Korea; 4 Department of Biomedical Engineering, National Yang-Ming University, Taipei, Taiwan, ROC

## Abstract

Bone is a flexible and electro active tissue that is vulnerable to various traumatic injuries. The self-healing of damaged bone tissue towards reconstruction is limited due to the lack of proper niche compliances. Nevertheless, the classical grafting techniques like autograft/allograft for bone repair pose challenges like bacterial infections and donor-site morbidity with unsatisfactory outcomes. The use of appropriate biomaterial with osteogenic potential can meet these challenges. In this regard, bioactive glass ceramics is widely used as a bone filler or graft material because of its bonding affinity to bone leading towards bone reconstruction applications without the challenge of post implant infections. Hence, the current study is aimed at addressing this potentiality of zinc (Zn) for doped the bioglass at nano-scale advantages for bone tissue repair. Since, Zn has been demonstrated to have not only antibacterial property but also the stimulatory effect on osteoblasts differentiation, mineralization by enhancing the osteogenic genes expression. In view of these, the present study is focused on sol–gel synthesis and pysico-chemical characterization of Zinc-doped bioglass nanoparticles (Zn-nBGC) and also analyzing its biological implications. The surface morphological and physiochemical characterizations using SEM, EDX, FT-IR and XRD analysis has shown the increased surface area of Zn-nBGC particles providing a great platform for biomolecular interaction, cytocompatibility, cell proliferation and osteogenic differentiation. The obtaining hydroxy apatite groups have initiated in vitro mineralization towards osteogenic lineage formation. Zn has not only involved in enhancing cellular actions but also strengthen the ceramic nanoparticles towards antibacterial application. Hence the finding suggests a biomaterial synthesis of better biomaterial for bone tissue engineering application by preventing post-operative bacterial infection.

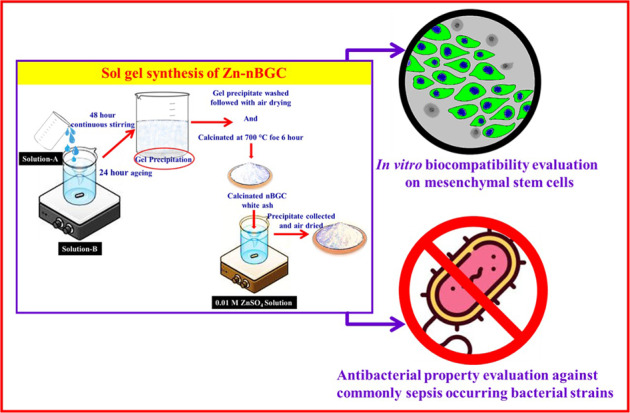

## Introduction

Bioglass ceramics are the most effective implant material applied in restorative dentistry and reconstruction of defective bone tissue [1, 2]. The bioceramics are preferred over metals and polymers in hard tissue reconstruction due to their superior chemical, thermal, dielectric and biological properties [3, 4]. Hence, the bio-ceramic based biomaterials have high potential and demand in the future for the reconstruction of‘ bone tissues [2]. The sol–gel method is a better procedure for the modification of surface composition as compared to (i) melt-derived and (ii) ion exchange procedures [4]. The sol–gel method involves low processing temperature and complete mixing of metal ions that aids in the formation of homogeneous nanostructures with high surface area [5–7]. Further, bio glass-ceramics are the mimetics of bioglasses they contain phosphate pentoxide (P_2_O_5_), as a common ingredient [8] also it possess greater biocompatibility, biodegradability, bioactivity and also osteoconductivity for which they are clinically applied in the restoration of skeletal system including bone, joints and teeth [6]. In orthopedic applications, the post implantation infection by microorganisms still stands with a great concern which requires incorporation of antibiotics additionally [7, 9]. Hence, there is need for the development of cost-effective multifunctional biomaterials. Earlier studies have shown that metallic particles such as silver (Ag), copper (Cu), zinc (Zn) and iron (Fe) to possess antibacterial property with positive effects on bone formation [10–12]. Among these metal ions, Zinc is a trace element which is present in the body (1.5–2.5 g and 85% of its present in bone and muscle) and responsible for many vital functions in the body. Zn is known for its non-toxicity as well as involving in various cellular mechanisms also [13]. Its other action involves as growth promoting effects on stem cells propagated for dental and orthopedic applications [14]. As per the previous reports, the role of zinc involves immune system functioning, cell division and skeletal development and therefore the Zn has been implemented into biomaterials for orthopedic and dental applications [15–17]. Besides all, Zn and Zn-based scaffolds provide mechanical properties to the mammalian bone cells and increased ECM mineralization in mesenchymal stem cell culture by stimulating the expression of ALP and osteopontin, which are the major factors for osteogenesis [18]. Some other additional studies have shown the osteogenic role of Zn on osteoblast like MC3T3-E1 cells collagen deposition and bone mineralization which are another major factors involved in osteoblastic behavior of the cells [19–21]. Hence the current study is focused on developing and evaluating the multifunctional (osteogenesis, angiogenesis and anti-bacterial) potential of zinc-doped bioglass nanoparticles (Zn-nBGC) to enhance the therapeutic benefits of the material for bone tissue engineering applications.

## Materials and methods

### Materials

Tetraethyl orthosilicate (TEOS), calcium nitrate, poly ethyl glycol (PEG), Diammonium hydrogen orthophosphate, ammonia, citric acid, ethanol (99.9%), ampicillin, Luria broth (LB), Mueller-Hinton (MH) agar, antibiotic and antimitotic solution, DAPI (4′,6-diamidino-2-phenylindole), FDA (fluorescein diacetate), alizarin red, trypsin-EDTA (ethylene diamine tetraacetic acid) and PBS were purchased from Hi-Media Chemical Co, USA. DMEM (Dulbecco’s Modified Eagle’s Medium), DMSO (Dimethyl sulfoxide), BSA (Bovine serum albumin),dexamethasone, β-glycerophosphate, MTT (3-[4,5-dimethythiazol-2-yl] 2,5-diphenyl tetrazolium bromide, Triton X-100, fetal bovine serum (FBS) were purchased from Sigma-Aldrich Co., St Louis, MO, USA. The C3H10T1/2 (mouse mesenchymal stem cells [mMSC]) used in this study were purchased from National Centre for Cell Science, Pune, India. The ATCC (American Type Culture Collection) bacterial strains, *Escherichia coli* (ATCC 25922), *Staphylococcus aureus*(ATCC 29213), *Klebsiella pneumonia* (ATCC 13883), *Pseudomonas aeruginosa* (ATCC15692), *Proteus mirabilis* (ATCC 29906), *Enterococcus faecalis* (ATCC 29212) and *Acetobacter aceti* (ATCC 15973), used for the antibacterial assessments were purchased from Hi-Media Chemical Co, USA [22, 23].

### Methods

#### Synthesis of nBGC and Zn-nBGC particles

The nano-bioglass ceramic nanoparticles with the compositional ratio of SiO_2_: CaO:P_2_O_5_ ~ 55:40:5 (Mol %) were synthesized via sol–gel method as reported earlier [24]. 0.5 g of nBGC was added to 0.01 M zinc sulfate solution and allowed for continuous stirring for 24 h. The nanoparticles were collected and rinsed with distilled water then air dried for overnight at 60 °C and finally subjected to physicochemical and biological activity analyses [25].

#### Physicochemical characterization

The surface morphology of synthesized nBGC and Zn-nBGC nanoparticles were investigated by scanning electron microscopy (SEM) imaging technique, where each of the samples were coated onto platinum surface and then scanned at 25 kV and 40 mA under vacuum. The hydrodynamic diameter and zeta potential of nBGC and Zn-nBGC nanoparticles were determined by using particle size analyzer (Malvern Zeta sizer nanosizer). The nBGC and Zn-nBGC nanoparticles were subjected for EDAX (Energy Dispersive X-ray analysis) analysis by applying JEOL-JEM2100F. Each sample was placed on carbon tape coated stub and smeared with platinum using JEOL JFC 1600 for 2 min at 10 mA. The peaks obtained as the EDAX spectrum correspond to the energy levels and referring to the individual elements present in each. Functional groups nBGC and Zn-nBGC nanoparticles were investigated with FT-IR spectrophotometrical analysis (American Perkin Elmer Co) by using KBr press spectral range from 2500 cm^−1^ to 400 cm^−1^. The resultant spectra of the individual samples were analyzed using OPUS software. To characterize the crystallinity of nBGC and Zn-nBGC nanoparticles, XRD patterns were obtained at room temperature using a (Panalytical XPERTPRO powder diffractometer) (CuKα radiation) operating at a voltage of 40 kV. The diffraction spectra were documented at 2θ with a range of 10–70° and scanned at a speed of 2° min^−1^. The presence of different phases in the nBGC and Zn-nBGC particles was identified by Hanawalt method using Philips X-pert high score software. The intensity of the diffracted pattern of the individual was calculated using Bragg’s law. The obtained 2θ values were compared with the JCPDS cards as standard.

#### In vitro protein adsorption

The amount of protein absorbed by nBGC and Zn-nBGC was measured following the reported procedure [26]. The pellets of nBGC and Zn-nBGC nanoparticles (each 250 mg) were immersed into 10% FBS containing medium at pH 7.4 for different durations 3, 6, 12, and 24 h. After the specified duration of immersion, the pellets were gently washed for the removal of unbound proteins and other residues and then individually placed into 1% SDS for 1 h with continuous agitation. This previous process was repeated thrice. Finally, the amount of protein adsorbed by the each individual pellets was estimated by Bradford method [26].

#### Hemocompatibility

The hemolytic potential of synthesized nBGC and Zn-nBGC in human red blood cells (hRBCs) was evaluated. RBC was collected from fresh blood sample and diluted with freshly prepared PBS. nBGC and Zn-nBGC of various concentrations (0.1, 0.2, 0.5, 1 and 2 mg/mL) were added to 500 μl of diluted RBC sample and incubated for 1 h. The samples were centrifuged at 1000 rpm for 3–5 min and supernatants were transferred into a fresh 96 well plate. The occurrence of lysis of hRBCs was measured by recording the absorbance at 570 nm [27].

#### Cytotoxicity assay

Murine mesenchymal stem cells [mMSC (C3H10T1/2)] were purchased from NCCS, Pune, India. In 24 well plate 1 × 10^6^ cells were seeded and incubated in 5% CO_2_. The seeded cells were treated with various concentrations (0.01, 0.02, 0.05, 0.1, 0.2, 0.5, 1 and 2 mg/mL) of nBGC & Zn-nBGC along with triton-X-100 as positive control and incubated for 24 h. After 24 h, the medium was removed and 500 μl of 0.5 % MTT solution was added into the each well followed by 4 h incubation. 100 μl of DMSO was added into each well and incubated for another2 h. The viable cells were determined by calorimetric analysis at 570 nm [28].

#### Cytocompatibility assessment

The cytocompatibility of nBGC and Zn-nBGC was examined with mMSC cells by fluorescein diacetate (FDA) and 4′,6′-diamidino-2- phenylindo (DAPI) staining. 1 × 10^6^ cells were treated in presence and absence of 0.2 mg/mL of nBGC and Zn-nBGC nanoparticles for 24 h. After the treatment period the cells were rinsed with PBS, incubated for 10 min with 5 μg/10 μL FDA solutions and subjected to microscopic examination. The cells were prefixed with 4% paraformaldehyde solution after the treatment period and staining solution (DAPI) in the concentration of 5 μg/10 μL was allowed to cover the cells for 15–20 min. The nuclei integrity of the cells was examined using fluorescent cell imaging system (EVOS FLoid Cell imaging station) [29].

#### Osteoblast differentiation and extracellular mineral deposition

Deposition of calcium on the matrix is hall mark for mineralization of differentiated osteoblast cells and it will be determined at cellular level by Alizarin red staining assay. Generally the calcium cations deposited in the extracellular matrix will react with alizarin reagent which will result in formation of red colored complex [30]. Briefly the 1 × 10^6^ of mMSCs were seeded and allowed to reach 70% confluent followed by the cells were incubated in presence and absence of nBGC and Zn-nBGC as well as normal and osteogenic medium for 21 days. Followed by the incubation period, the cells were fixed with 10% formalin and rehydrated. Finally, the cells were stained with 1% of alizarin red solution (pH- 4) to determine the calcified nodules formation under microscopic observation. The quantitative analysis of calcium formation was also determined by colorimetric analysis. Briefly the nodules were dissolved with 1 N acetic acid and subjected to spectrophotometric analysis at 570 nm [24].

#### Antibacterial activity

The post implantation infection associated the orthopedic implants were challenge in the field of bone tissue engineering. The antibacterial activity of the synthesized nBGC and Zn-nBGC nanoparticles were evaluated by zone of inhibition and Minimum Inhibitory Concentration (MIC) procedures against various osteo-sepsis associated bacterial strains such as *Escherichia coli, Staphylococcus aureus, Klebsiella pneumonia, Pseudomonas aeruginosa, Proteus mirabilis and Enterococcus faecalis*. Briefly, 2.5 μl of mother culture was inoculated into 5 mL of LB broth along with various concentrations (0.1, 0.2, 1 and 2 mg/mL) of nBGC and Zn-nBGC nanoparticles for overnight incubation [31]. After the incubation period the inoculated strains were subjected to determine the biomass density by spectrophotometric evaluation at 600 nm. In addition the bacterial growth inhibition was also confirmed by zone of growth inhibition method. Briefly 100 μl of overnight incubated mother culture was spread on LB agar plates individually and wells were pricked with the diameter of 50 mm. In each well the nBGC and Zn-nBGC particles were loaded and subjected for overnight incubation. After the incubation period the diameters of the zones formed were measured along with the well diameter to determine the growth of bacterial on the surrounding zone of the nBGC and Zn-nBGC particles loaded in the wells.

#### Biofilm eradication

The formation of biofilm (accumulation of microorganisms) is a lead to a variety of dental diseases. Hence the biofilm eradication potentiality of the Zn-nBGC particles was assessed using common usual pathogens such as Staphylococcus aureus (ATCC 29213), *Pseudomonas aeruginosa* (PAO1) and *Acetobacter aceti* (ATCC 15973) obtained from ATCC. The 100 μL of each freshly cultured bacterium into MHB media supplemented with 0.2% glucose were cultured in 96-well plate and incubated at 37 °C, for 24 h in order to form biofilm. The wells containing biofilm were washed thrice with sterile PBS. Various concentrations (0.1, 0.25, 0.5 and 1 mg/mL)of nBGC and Zn-nBGC particles were incubated with bacterial plates containing biofilms in addition the sterile PBS commercially available antibiotic (Ampicillin) were used as negative and positive controls respectively followed by the plates were incubated in a shaking incubator for 24 h at 37 °C. After the incubation period the medium was discarded and wells were washed with PBS for twice finally subjected to air-dry for 1 h under sterile condition. The percentage of biofilm eradiated from each bacterial cultures was quantified by measuring the absorbance after applying crystal violet stain as previously described [31, 32].

### Statistical analysis

All the experiments were performed in triplicates and the results were expressed as mean ± S.D. The statistical significance was analyzed by Student’s *t* test. A *p* value < 0.05 was considered as statistically significant.

## Results and discussion

### Morphological and surface characterization of nBGC & Zn-nBGC

**Fig. 1:**
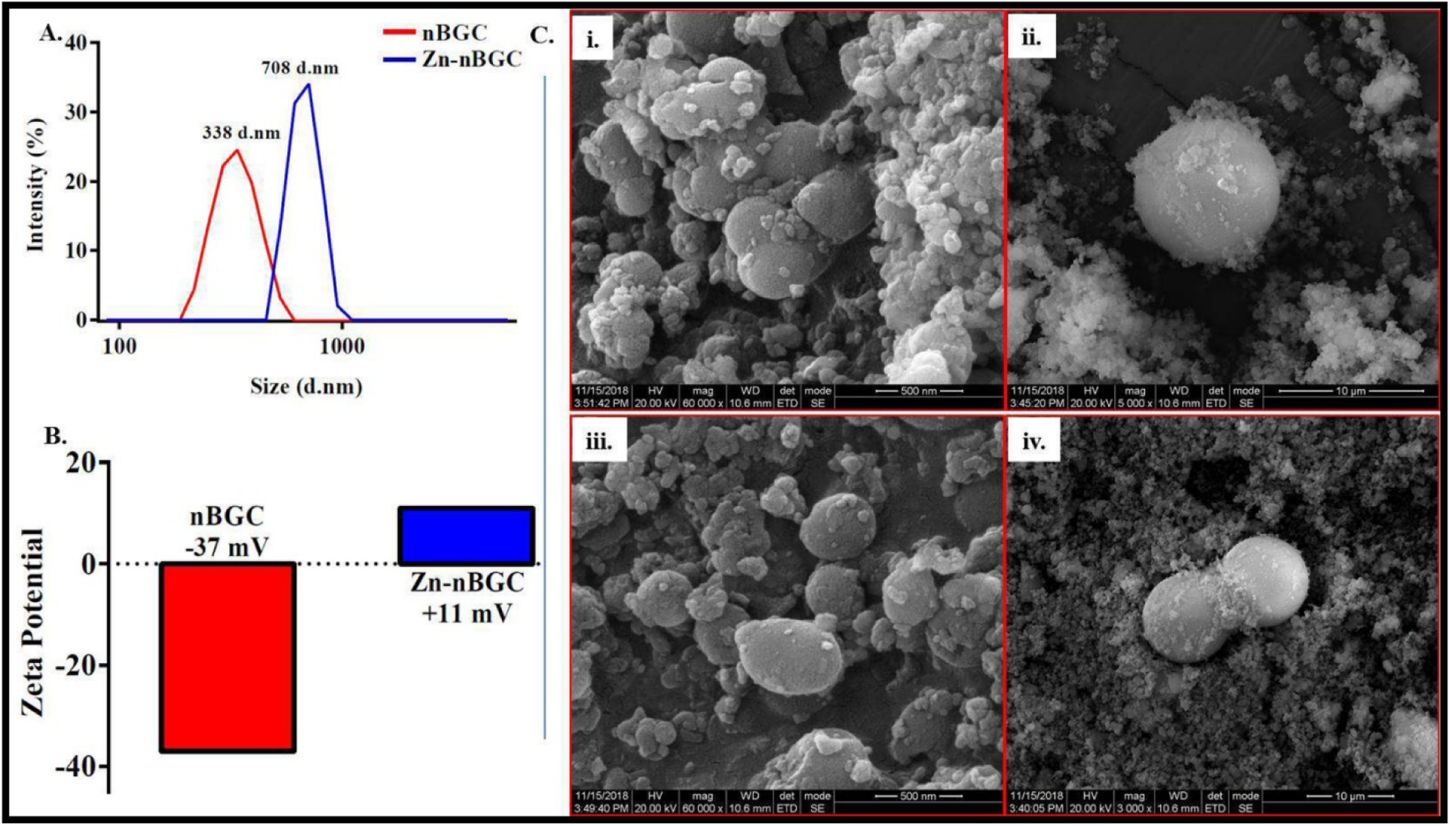
**A** DLS Spectra of nBGC & Zn-nBGC representing the hydrodynamic diameter at 338 d.nm & 708 d.nm respectively. **B** Surface charge of the synthesized nBGC and Zn-nBGC particles are determined by means of Zeta potential (mV) found at −37 mV for nBGC due to the acidic nature of it. **C** SEM image of nBGC (i, ii) and Zn-nBGC (iii, iv) at different magnification showing circular structures of nanoparticles with a glossy surface

**Fig. 2:**
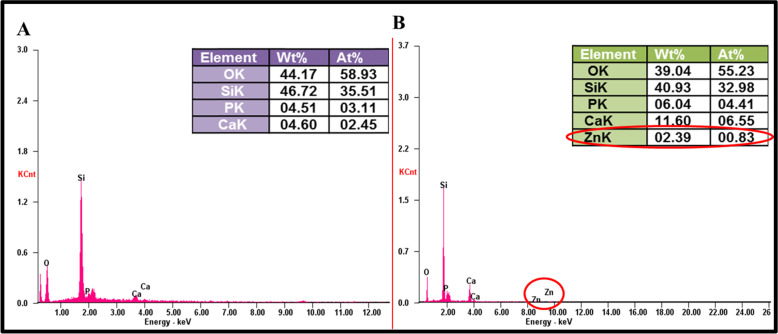
EDAX spectra represents the presence of major elements in synthesized nBGC (**A**) and Zn-nBGC (**B**) nanoparticles

Synthesis of Zn-nBGC by doping Zn ion onto nBGC nanoparticles was carried out by sol–gel method. The synthesized nanoparticles were subjected to physio-chemical characterization by various processes. The hydrodynamic diameter of nBGC and Zn-nBGC nanoparticles were measured by DLS spectral analysis were found to beat 338 d.nm and 708 d.nm, respectively as given in Fig. 1A. The increased diameter of Zn-nBGC is possibly due to the aggregation of Zn metal on nBGC surface and the obtained broad spectra also attribute to the formulation of poly-dispersed nanoparticles. Surface charges of the individual nanoparticles was determined by the Zeta potential (mV) analysis and shown in Fig. 1B. The Zeta potential was at −37 mV for nBGC and +11 mV for Zn-nBGC. The cationic nature of Zn-nBGC might be due to the aggregation of Zn^+2^ ion on nBGC surface. The morphology of the formulated nanoparticles was analyzed by SEM analysis which revealed bulbous like nanoparticles formation with glassy phase due to the presence of silica [24]. The rod like structure observed on the surface of the circular nBGC nanoparticles is presented in Fig. 1C (i, ii). It confirms the presence of aggregated Zn^2+^ on the surface of it (Fig 1C iii, iv). It also further indicates the presence of heterogeneous nanoparticles. The compositional analysis by EDAX spectrum presented in Fig. 2 revealed the presence the key elements such as calcium (Ca), phosphate (PO4) and silica (Si) (Fig. 2A) in addition to the presence of Zn atoms (2.39 Wt %) found in Zn-nBGC (Fig. 2B).

### Chemical-molecular characterization

**Fig. 3:**
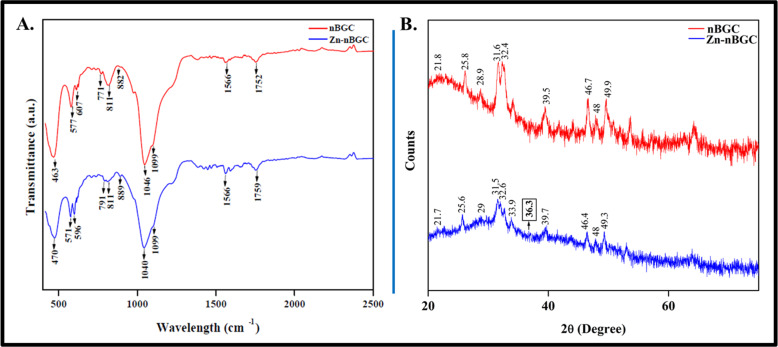
**A** FT-IR Spectra of nBGC & Zn-nBGC confirming the presence of molecular bonding and stretching with corresponding peaks respectively. **B** XRD Spectra of nBGC & Zn-nBGC confirming the crystalline nature of nBGC and Zn-nBGC respectively

The FTIR spectra of both nBGC and Zn-nBGC particles represented in Fig. 3A, showing the transmittance bands in the range of 400–2500 cm^−1^. The respective spectra consisting the combination silicate, phosphate, calcium and carbon groups are contributed for the construction of the ceramic nanoparticles [33]. The major bands conforming the presence of silicate groups are observed at around 470 cm^−1^, 600 cm^−1^attributing the Si–O bending and O–Si–O bending respectively [34–36]. Additionally Si–O–Si symmetric stretching is obtained at 771 cm^−1^ and 791 cm^−1^ for nBGC and Zn-nBGC respectively due to the of oxygen bridging between tetrahedrals [35]. Si–O–Si asymmetric stretching (900–1100 cm^−1^) and Si–O stretching (860–940 cm^−1^) bands with non bridging are accepted to be a characteristic of silicate network since SiO_2_ is present as a major building constituent [34–36]. The non-bridging oxygen atoms have caused peaks appeared at 860–940 cm^−1^ representing Si–O stretching [33, 35]. C–O vibration is found in the range of 800–890 cm^−1^ attributing the presence of carbonated group of hydroxy apatite [36].The crystalline phosphate group is determined by the P–O bending found around 570 cm^−1^ and the vibration around 1099 cm^−1^ representing the presence of PO_4_^3-^ indicating the formation of hydroxyl carbonate apatite (HCA) layer [37, 38]. This further confirms the crystalline nature of nBGC and Zn-nBGC. Collectively, the IR spectral data indicate that the addition of inert Zinc ion to nBGC does not modify the crystalline properties of nBGC. However, there was slight shift in peak observed in Zn-nBGC, because the presence of ZnO could enhance the chemical stability of silicate glasses [36].

The phase characteristics of nBGC and Zn-nBGC nanoparticles were assessed by crystalline XRD spectral analysis. As per previous reports the crystalline nature of the synthesized nanoparticles were confirmed by the peaks seen in Fig. 3B. The highest peak at 25.8° (JCPDS @ 76-0694), 31.5°(JCPDS @ 09-0432), 32.6° (JCPDS @ 76-0694) conforms the hydroxyapatite [Ca_10_(PO_4_)_6_(OH)_2_](JCPDS @ 09-0432) [38, 39]. CaSiO_3_ phase was determined by the small peak appeared at 21.8° (JCPDS @ 10-489) [24]. Also the difractogram analysis revealed the formation of silicate crystalline ceramic phase by the presence of Na_2_CaSi_2_O_6_ (JCPDS # 01-077-2189) [40]. The indicated peak at 36.3° conforms the presence of zinc (Zn) plane as a content of Zn-nBGC [41]. The peaks at 28.9° and 48° in nBGC is found to be decreased in Zn-nBGC spectra at 29° and 48° corresponding to Calcite (CaCO_3_) (JCPDS @ 88-1809) [38]. It is expected due to the substitution of Ca^2+^ ion with Zn-nBGC which is a major element of calcite.

### In vitro protein adsorption

**Fig. 4:**
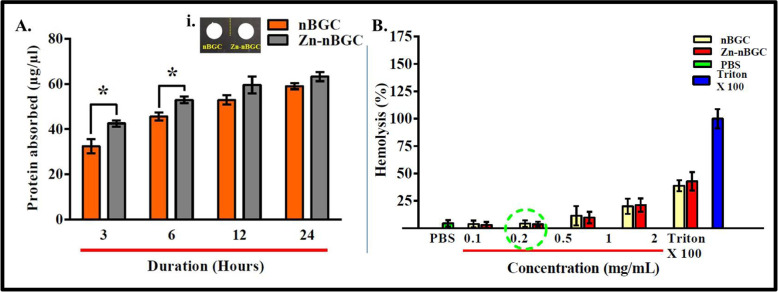
Protein adsorption studies of nBGC and Zn-nBGC at various durations (3, 6, 12 and 24 h). **A** (i) The pellets of nBGC and Zn-nBGC. **B** Hemolysis analysis showing the increased nBGC and Zn-nBGC concentration resulting in higher lysis of RBCs. ‘*’ Indicates the significant difference compared to the control where *P < 0.05

The tissue engineering constructs in vivo implantation adsorb a variety of key proteins including fibronectin, vitronectin and other signaling factors on their surface from the surrounding body fluids which possess a crucial role in regulating cell attachment and differentiation [42–44]. The amount of absorbed protein at different time points of incubation of nBGC and Zn-nBGC pellets with DMEM with FBS is shown in Fig. 4A. Zn-nBGC adsorbed more protein as compared to nBGC pellet as shown in Fig. 4A at 3 and 6 h durations. Generally the silanol group present in silica based biomaterials is vital for protein adsorption [26].The increase in protein adsorption observed at initial time points of incubation (3 h and 6 h) was not seen at later time points (12 h and 24 h) due to the attainment of saturation. The saturation of protein absorption may be due to the exposure of more hydrophilic moieties on the surface of Zn-nBGC nanoparticles.

### Hemocompatibility

The non-compatible implants may lead to rupture or lysis of red blood cells within the circulation in vivo. Hence, the hemocompatibility of the Zn-nBGC and nBGC particles were assessed at various concentrations (0.1, 0.2, 0.5, 1 and 2 mg/mL) incubated with human red blood cells (hRBCs). The increase in the concentration of particles was usually found to promote more rupture of RBC’s (Fig. 3B). Generally, up to 0.2 mg/ mL concentration of Zn-nBGC or nBGC particles there was no significant toxicity. The level of toxicity is corroborated with the clinical reference value of haptoglobin level [0.5–2.2 g/L (SI units)]. The haptoglobin is an acute-phase reactant whose principal clinical utility is in defining conditions of hemolysis. An increase in the haptoglobin level will lead to infection and inflammation.

### Cytotoxicity and cytocompatibility

**Fig. 5:**
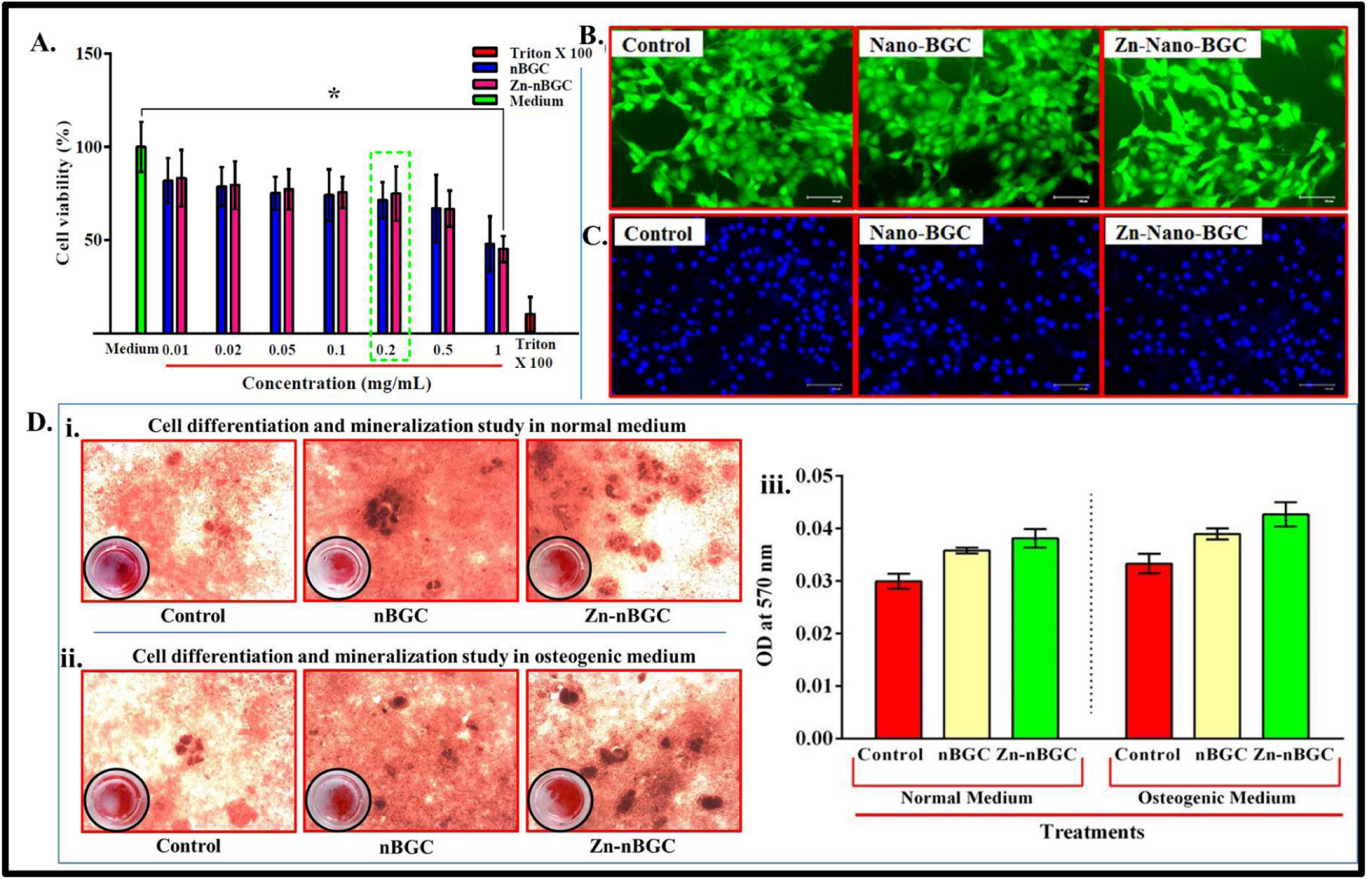
Cell viability (%) of BGC and Zn-nBGC determined by MTT assay in mouse mesenchymal stem cells (C3H10T1/2) at various concentrations after 24 h treatment. ‘*’ Indicates the significant difference compared to the control where *P < 0.05 (**A**). Cytocompatibility of nBGC and Zn-nBGC on C3H10T1/2 at the concentration of 0.2 mg/ml by FDA and DAPI staining showed the cytocompatibility (**B** and **C**). Osteoblast differentiation and mineral deposition study performed on C3H10T1/2 by alizarin red staining assay (**D i, ii**) and the quantitative analysis of biomeneralization showed enhanced biomineralization in presence of osteogenic stimulants (**D iii**)

Prior to their use in bone tissue engineering, the cytocompatibility of the nanoparticles must be assessed to determine whether they are cytotoxic to cells. Generally, the metabolically active cells are considered as viable cells. The cell viability is determined by measuring the amount of purple colored formazan crystals corresponding to the viable cell calorimetrically by MTT assay [45]. Cytotoxic activity of nBGC and Zn-nBGC of determined by MTT assay in mouse mesenchymal stem cells (C3H10T1/2) after 24 h treatment (Fig. 5A) the metabolic activity of the cells were found to be more up to the concentration of 0.2 mg/ mL whereas, increasing the concentrations of nanoparticles were found to increase the toxicity (Fig. 5A). It might be due to the alkalinity which would have generated due to burst ionic dissolution of calcium and silicon ions in the aqueous environment, causing hyperosmotic condition leading to cell shrinkage and cell death through membrane transporters on their respective osmolytes [30, 46]. FDA and DAPI results with optimal concentration of particles (0.2 mg/mL) was shown the spreading of mMSC and intact nucleus by DAPI staining (Fig. 5B, C) found that the particles at particular concentration is cytocompatible for tissue engineering applications.

### Osteoblast differentiation and extracellular mineral deposition

Extracellular mineralization in terms of calcium and phosphate deposition competency of nBGC and Zn-nBGC nanoparticles was determined by Alizarin Red staining method as calcium and phosphate are the late-stage markers of osteoblastic persistence in the sample [47, 48]. Calcium deposition mediated by nBGC and Zn-nBGC nanoparticles towards differentiation of mouse mesenchymal stem cells (C3H10T1/2) into osteoblast cells were performed after 21 days of culture cells in presence and absence of nBGC and Zn-nBGC as well as normal and osteogenic medium (100 mM Dexamethasone, 10 mM β-glycerophosphate and 50 g/mL Ascorbic acid supplemented with DMEM medium). The result shows that (Fig. 4D i, ii) higher number dark red-maroon patches in Zn-nBGC treated cells in osteogenic medium compare than other treatments. In addition the quantitative analyses of the deposited minerals are analyzed by measuring the OD at 570 nm is represented graphically (Fig. 4D iii). It was found that enhanced mineralization efficacy of Zn-nBGC than nBGC nanoparticles in presence of osteogenic medium than normal medium. It is indicating that the integration of Zn metal ion actively the osteoconductive properties of nBGC particles.

### Antibacterial activity

**Fig. 6:**
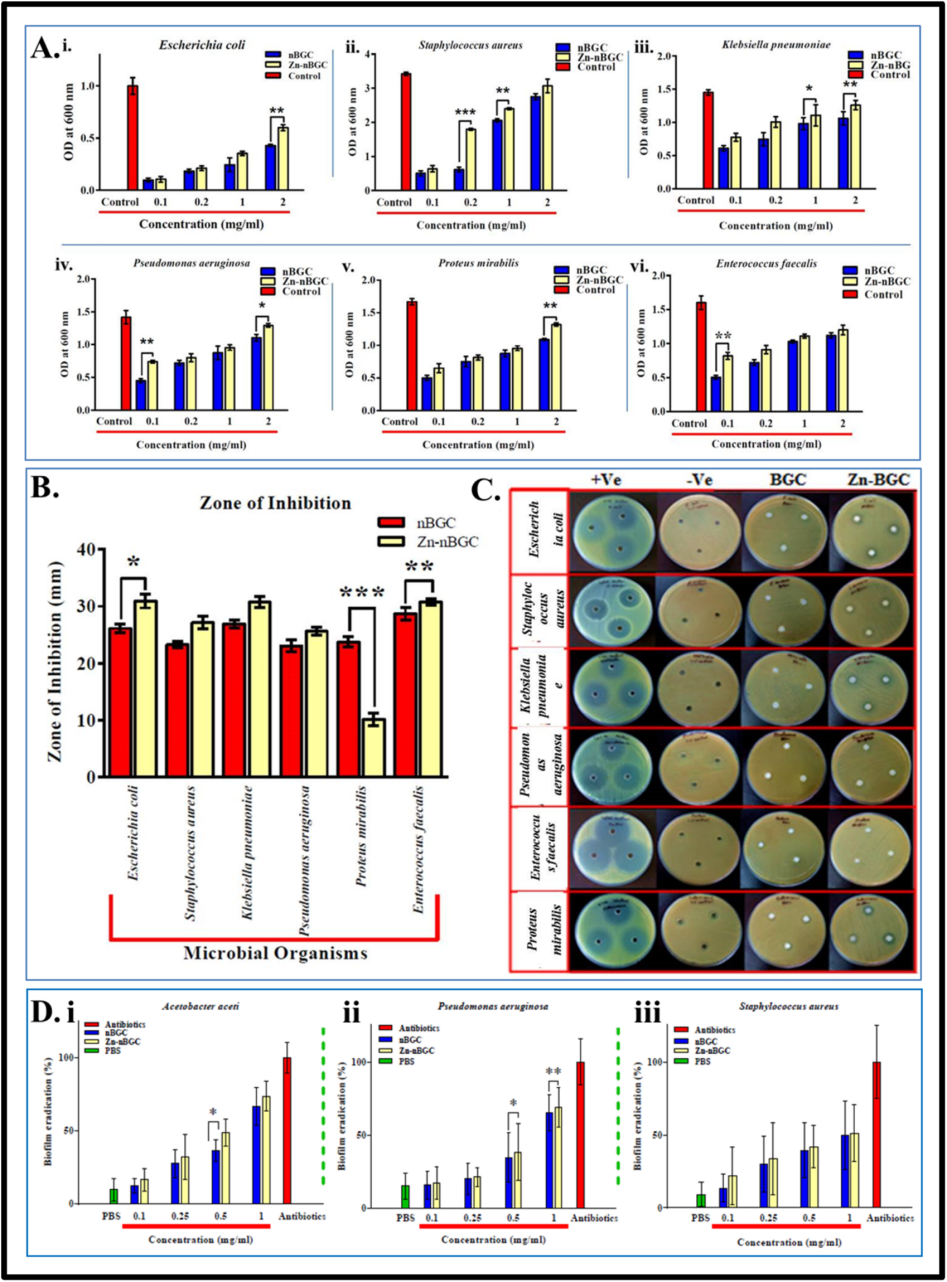
**A** Minimum inhibitory concentration. **B** Measured zones formed by the effect of nBGC and Zn-nBGC examined against clinical bacterial isolates of (i) *Escherichia coli*, (ii) *Staphylococcus aureus*, (iii) *Klebsiella pneumoniae*, (iv) *Pseudomonas aeruginosa*, (v) *Proteus mirabilis and* (vi) *Enterococcus faecalis* bacterial strains after 24 h incubation. **C** Pictorial representation of zone of inhibition assay performed on respective organisms. **D** Biofilm eradication potentiality of nBGC and Zn-nBGC against ATCC strains of (i) *Acetobacter aceti*, (ii) *Pseudomonas aeruginosa* and (iii) *Staphylococcus aureus*.‘*’ Indicates the significant difference compared to the control where ****p < 0.0001, ***p < 0.001, **p < 0.01 and *P < 0.05

Antibacterial properties of nBGC and Zn-nBGC nanoparticles were evaluated in ATCC bacterial strains (*Escherichia coli, Staphylococcus aureus, Klebsiella pneumoniae, Pseudomonas aeruginosa, Proteus mirabilis and Enterococcus faecalis*) against PBS as negative control and commercial ampicillin antibiotics as positive control. The Minimal Inhibition Concentrations (MIC) of nBGC and Zn-nBGC were evaluated at various concentrations (0.1, 0.2, 1, 2 mg/mL). The results (Fig. 6A) determine the growth inhibitory potentiality on increasing the concentration of nanoparticles [31]. After the overnight incubation of each individual bacterium plates treated with test (nBGC and Zn-nBGC) and control samples, growth inhibited zones are appeared and the diameter of the zones were compared (Fig. 6B). Figure 6C shows the better inhibitory effect of Zn metal doping compared to nBGC. Microbial biofilms, which are elaborate and highly resistant microbial aggregates formed on surfaces or medical devices, cause two-thirds of infections and constitute a serious threat to public health [49]. Since, biofilm formation is great challenge for any therapeutic implant agents to avoid post implant infections. Figure 6D shows the biofilm eradication potential of nBGC and Zn-nBGC against the most prominent biofilm forming ATCC strains of (i) *A aceti*, (ii) *P aeruginosa* and (iii) *S aureus*. The assay, performed at increasing concentrations of nBGC and Zn-nBGC nanoparticles (0.1–1 mg/mL) along with the conventional antibiotic ampicillin. As illustrated in Fig. 6D, Zn-nBGC showed improved anti-biofilm activity compared to nBGC indicating addition of zinc enhanced the anti-biofilm properties of Bioglass particles. Notably, ~50% eradication of preformed biofilms was observed against *A. aceti* at 0.5 mg/mL of Zn-nBGC treatment and ~30–40% reduction was observed against *P. aeruginosa* and *S. aureus* biofilms. Whereas there was no significant reduction observed at selected optimal concentration (0.2 mg/mL) Table 1.

**Table 1:** Mean and SE of measured diameter of observed zones along with 55 mm well diameter formed by nBGC and Zn-nBGC on *Escherichia coli, Staphylococcus aureus, Klebsiella pneumoniae, Pseudomonas aeruginosa, Proteus mirabilis and Enterococcus faecalis*

Bacterial Strains	BGC	Zn-BGC
*Escherichia coli*	26.15 ± 0.204	30.94 ± 0.208
*Staphylococcus aureus*	23.3 ± 0.082	27.15 ± 0.139
*Klebsiella pneumoniae*	26.95 ± 0.04	30.8 ± 0.278
*Pseudomonas aeruginosa*	23.08 ± 0.42	25.64 ± 0.27
*Proteus mirabilis*	23.8 ± 0.274	10.17 ± 0.143
*Enterococcus faecalis*	28.67 ± 0.27	30.77 ± 0.102

## Conclusion

The present study has described the synthesis and characterization of zinc metal doped nano-bioglass ceramics (Zn-nBGC) and also evaluated the osteogenic potentiality of it towards bone tissue regeneration. The crystalline physiology has enhanced the biological ability along with antibacterial property of the nanoparticles. In vitro assessments such as hemocompatibility against hRBCs, cytotoxicity, cytocompatibility, biocompatibility and also mineral deposition studies in mammalian cell lines have evidenced the enhanced osteogenic effect of Zn-nBGC, as compared to nBGC. The enhanced bone regenerative property has also verified by the presence of bone like hydroxyapatite in the nanoparticles. At lower concentration there was no toxic effect. However higher concentration and longer incubation of the nanoparticles resulted in increase of toxicity.
